# Cardiovascular disease risk differences between bus company employees and general workers according to the Korean National Health Insurance Data

**DOI:** 10.1186/s40557-018-0242-z

**Published:** 2018-05-08

**Authors:** Ji-Hoo Yook, Dong-Wook Lee, Min-Seok Kim, Yun-Chul Hong

**Affiliations:** 1 Department of Preventive Medicine, Seoul National University College of Medicine, Seoul, Republic of Korea; 2 Institute of Environmental Medicine, Seoul National University Medical Research Center, Seoul, Republic of Korea

**Keywords:** Bus company employees, Cardiovascular disease, Commercial driver, National health insurance

## Abstract

**Background:**

Bus drivers are known to be highly at risk of cardiovascular diseases. In this study, we assessed the cardiovascular disease prevalence of bus company employees in Seoul, South Korea, and compared the results to those of general workers.

**Methods:**

We analyzed the 2014 Korean National Health Insurance (NHI) data and defined hypertension, diabetes, dyslipidemia, ischemic heart disease, and cerebrovascular disease based on the KCD-6 medical diagnoses. We used bus company employees as surrogate participants of bus drivers due to the characteristics of Korean NHI data. We identified bus company employees in Seoul based on one’s workplace which the insurance is registered. The prevalence of five diseases was compared between the bus company employees and general workers. We also calculated the odds ratios (OR) of five diseases between the bus company employees and general workers. To compensate the vast demographical differences between the two groups, we performed propensity score matching.

**Results:**

Bus company employees have higher OR for having hypertension (OR 1.33, 95% CI: 1.28–1.39), diabetes mellitus (1.14, 95% CI: 1.08–1.22), and dyslipidemia (1.23, 95% CI: 1.17–1.29) than the general workers or propensity score matched controls. However, the OR of having ischemic heart disease were not significant. The OR of cerebrovascular disease were lower in bus company employees than in the general workers after adjusting the covariates, but similar in the propensity score matched model.

**Conclusion:**

This study showed that the ORs of cardiovascular disease risk factors are high in bus company employees when compared to the general working population. Further studies with the longitudinal design should be conducted to confirm the causal association.

## Background

Cardiovascular diseases and related complications are the leading causes of death worldwide and are projected to gradually increase in the near future [1]. Occupation is one of the established risk factors of cardiovascular diseases and death [2]. Confirmed work-related cardiovascular disease cases may be associated with worker’s compensation [3]. In 2014, 355 workers were compensated for a work-related disease due to cardiovascular and neurovascular diseases in Korea [4].

Commercial drivers are known to carry diverse health problems, such as cardiovascular diseases. Their health problems may be more prevalent and severe than those in other types of occupation [5]. Long working hours, shift work, cabin ergonomic factors, loud noise, carbon monoxide, chemical materials, social isolation, and lack of decision-making by the authority are occupational health risk factors of commercial driving [5, 6]. Work-related diseases of commercial drivers have been studied in various aspects. The incidence of various diseases like bladder cancer, musculoskeletal diseases, depressive disorder, post-traumatic stress disorders, and cardiovascular diseases is higher in commercial drivers compared to other occupations [7–10]. Important risk factors of cardiovascular events, such as hypertension, diabetes mellitus, and obesity, are also more prevalent in commercial drivers [11–15].

Drivers who carry passengers are highly at risk of stroke than drivers carrying goods [16]. Bus drivers’ cardiovascular health is closely related to public safety and is of high concern because the bus carries relatively large numbers of passenger at once. Being an exception of the labor standard act that prevents workers from working over 12 h a day in Korea, investigations on bus drivers in South Korea showed long working hours, which is a considerable risk factor of cardiovascular diseases [17].

However, studies on the actual health status of a relatively large group of commercial bus drivers were limited. We planned to determine cardiovascular disease prevalence and their risks on bus drivers and compared the results to that of general workers using Korean National Health Insurance data. We analyzed bus company employees’ data as surrogate participants of bus drivers because the Korean National Health Insurance data contain that of worker’s company only and we cannot determine one’s actual job characteristics in the company. This procedure can be admitted as a majority (over 90%) of bus company employees is bus drivers [18]. If we can find significant differences in the disease prevalence between bus company employees and general workers, this study may help prevent bus drivers from suffering work-related cardiovascular disease.

## Methods

### Data source

We used Korean National Health Insurance (NHI) data for this study. The National Health Insurance is mandatory for all residents in Korea and covers 100% of the Korean population [19, 20]. All members are categorized into three groups: employees or employees’ dependent family group, self-employed and his or her dependent family group, and medical aid beneficiary group [19]. The National Health Insurance System now offers four main databases: qualification and contribution, health insurance claim, health check-up, and long-term care insurance data [20, 21]. We used the 2014 data that was the latest one provided at the time of the study. We merged and analyzed the first three database using the data of the employee group only.

### Study participants

We defined that any person who registered the National Health Insurance under the city bus companies in Seoul, Korea, since 2014 is a bus company employee. The total number of target city bus companies in Seoul was 65. We defined the general working group as everybody who were registered in the NHI under any company at the same time. We excluded those who did not receive biennial general health check-up in 2014. A total of 15,719 bus company employees and 8,033,907 general workers were enrolled.

### Disease definition

We regarded that one has specific disease if the NHI claim outpatient records and hospital admissions contain any of the specified KCD-6 codes in its main and secondary diagnosis field one or more times in 2014. We listed the codes of hypertension, diabetes mellitus, dyslipidemia, ischemic heart disease, cerebrovascular disease in the Table 1.

**Table 1:** KCD-6 codes used in disease definition

Disease	Diagnosis	KCD-6 Code
Hypertension	primary (essential) hypertension	I10
	hypertensive heart disease	I11
	hypertensive renal disease	I12
	hypertensive heart and renal disease	I13
Diabetes mellitus	non-insulin-dependent diabetes mellitus	E11
Dyslipidemia	disorders of lipoprotein metabolism and other lipidemias	E78
Ischemic heart disease	angina pectoris	I20
	acute myocardial infarction	I21
	subsequent myocardial infarction	I22
	certain current complications following acute myocardial infarction	I23
	other acute ischemic heart diseases	I24
	chronic ischemic heart disease	I25
Cerebrovascular disease	subarachnoid hemorrhage	I60
	intracerebral hemorrhage	I61
	other non-traumatic intracranial hemorrhage	I62
	cerebral infarction	I63
	stroke, not specified as hemorrhage or infarction	I64
	occlusion and stenosis of precerebral arteries, not resulting in cerebral infarction	I65
	occlusion and stenosis of cerebral arteries, not resulting in cerebral infarction	I66
	other cerebrovascular diseases	I67
	cerebrovascular disorders in diseases classified elsewhere	I68
	sequelae of cerebrovascular disease	I69

### Cardiovascular risk factor definition

We extracted data from the general health check-up database to define cardiovascular risk factors, such as obesity, lack of exercise, smoking, and heavy drinking. Obesity is defined as having a body mass index (BMI, weight (kg)/height (m)^2^) of > 25. Lack of exercise is defined as performing moderate to high-intensity exercises in < 3 times a week. We regarded a participant as a smoker if he or she answered smoking status question as a current smoker. Heavy drinking is defined as a person who reportedly drink > 7 glasses of alcoholic beverage in a week.

### Propensity score matching

We used propensity score matching to define a comparison group. The comparison group was from the general working group. The propensity score is defined as the inverse subject probability of receiving a treatment or being in a certain condition. The propensity score is estimated by using a multinomial logistic model on confounding factors between treatment and outcome [22, 23]. In this case, the condition is currently working in a bus company. Then, the propensity score (matched, adjusted, or weighted) was considered to estimate the distribution of effects in treated and untreated subjects [22–24].

We performed a logistic regression to estimate the propensity score. We included seven variables, i.e., sex, age group, income level, obesity, smoking, heavy drinking, and lack of exercise in the score model. For every person in the bus company employee group, three persons with the most similar propensity score were selected from the general working group. We used greedy matching algorithm with eight digits. We chose one to three matching because one to four matching yielded statistically significant different characteristics in some variables between two groups while one to three matching showed no significantly different characteristic.

### Data analysis

We calculated the prevalence of each disease in the bus company employee’s and general working group. The odds ratios (ORs) of five diseases were calculated for both the bus company employee and general working group. The logistic regression analysis was performed using the following two models: a crude model and an adjusted model which used age groups, sex, income level quartile, and cardiovascular risk factors such as obesity, lack of exercise, smoking status, and heavy drinking as covariates. We calculated the ORs of five diseases for bus company employee group and propensity score matched group using chi-squared tests. SAS 9.4 and Microsoft Excel 2016 were used for all statistical analyses. The significance level was set to *p*-value of < 0.05.

## Results

Table 2 shows the general characteristics of bus company employees and general workers. About 96.3 and 64% of the bus company employee and general working groups were males. The mean age of bus company employees and general workers was 50.3 and 42.1 with the standard deviation of 7.30 and 11.50, respectively. About 83.2% of the bus company employees were in the third quartile based on the income level. Obesity were more prevalent in the general working group than in the bus company employee group, with 66.3 and 40.8% obese individuals in each group, respectively. About 23.9 and 53.3% of the bus company employee and general working groups were active smokers; 19.8 and 17.0% of bus company employees and general workers were heavy drinkers, respectively; and 37.6 and 61.3% of the bus company employee and general working groups lack adequate exercise, respectively. All seven characteristics were different between two groups with the *p*-value below 0.0001.

**Table 2:** General characteristics of bus company employees and general workers

		Bus company employees		General workers		
		n	%	n	%	*p*-value
Total		15,719	100.0	8,033,907	100.0	
Sex	Male	15,130	96.3	5,139,814	64.0	< 0.0001
	Female	589	3.8	2,894,093	36.0	
Age	15–29	52	0.3	1,242,608	15.5	< 0.0001
	30–39	1088	6.9	2,338,147	29.1	
	40–49	5656	36.0	2,227,686	27.7	
	50–59	7638	48.6	1,668,020	20.8	
	> 60	1285	8.2	557,446	6.9	
Income level	25%P	181	1.2	1,805,809	22.5	< 0.0001
	50%P	640	4.1	1,852,773	23.1	
	75%P	13,072	83.2	2,135,139	26.6	
	100%P	1826	11.6	2,240,186	27.9	
Obesity^a^	yes	6410	40.8	2,708,099	33.7	< 0.0001
	no	9309	59.2	5,325,808	66.3	
Smoking	yes	3756	23.9	4,282,941	53.3	< 0.0001
	no	11,963	76.1	3,750,966	46.7	
Heavy drinking^b^	yes	3104	19.8	1,362,534	17.0	< 0.0001
	no	12,615	80.3	6,671,373	83.0	
Lack of exercise^c^	yes	5904	37.6	4,923,386	61.3	< 0.0001
	no	9815	62.4	3,110,521	38.7	

^a^Obesity is defined as having a body mass index of above 25 kg/m^2^

^b^Heavy drinking is defined as a person who reportedly drink > 7 glasses of alcoholic beverage in a week

^c^Lack of exercise is defined as performing moderate to high-intensity exercises in < 3 times a week

Table 3 shows the general characteristics of bus company employees and propensity score matched controls. A total of 47,250 controls were selected from the general working groups. The characteristics between the two groups were similar, as their characteristics were matched as close as possible.

**Table 3:** General characteristics of bus company employees and propensity score matched controls

		Bus company employees		Matched Controls(1:3)		
		n	%	n	%	*p*-value
Total		15,719	100.0	47,257	100.0	
Sex	Male	15,130	96.3	45,402	96.3	0.8841
	Female	589	3.8	1755	3.7	
Age	15–29	52	0.3	152	0.3	0.9999
	30–39	1088	6.9	3268	6.9	
	40–49	5656	36.0	16,968	36.0	
	50–59	7638	48.6	22,914	48.6	
	> 60	1285	8.2	3855	8.2	
Income level	25%P	181	1.2	573	1.2	0.9099
	50%P	640	4.1	1888	4.0	
	75%P	13,072	83.2	39,230	83.2	
	100%P	1826	11.6	5466	11.6	
Obesity^a^	yes	6410	40.8	19,254	40.8	0.9179
	no	9309	59.2	27,903	59.2	
Smoking	yes	3756	23.9	11,234	23.8	0.8542
	no	11,963	76.1	35,923	76.2	
Heavy drinking^b^	yes	3104	19.8	9346	19.8	0.8533
	no	12,615	80.3	37,811	80.2	
Lack of exercise^c^	yes	5904	37.6	17,742	37.6	0.8941
	no	9815	62.4	29,415	62.4	

^a^Obesity is defined as having a body mass index of > 25 kg/m^2^

^b^Heavy drinking is defined as a person who reportedly drink > 7 glasses of alcoholic beverage in a week

^c^Lack of exercise is defined as performing moderate to high-intensity exercises in < 3 times a week

Table 4 presents five cardiovascular diseases’ prevalence between the bus company employees and general workers and propensity score matched controls. The prevalence of all five diseases was higher in the bus company employee group than that in the general working group. The prevalence of hypertension, diabetes, and dyslipidemia was higher in the bus company employee group than in the matched control group. However, the prevalence of cerebrovascular disease was lower in the bus company employee group than that in the matched control group. The prevalence of ischemic heart disease was insignificantly different between the two groups (*p*-value 0.7529).

**Table 4:** Cardiovascular disease prevalence of bus company employees and that of general workers and matched controls

	Bus company employees		General workers			Matched Controls		
	(*n* = 15,719)		(*n* = 8,033,907)			(*n* = 47,257)		
	n	%	n	%	*p*-value	n	%	*p*-value
Hypertension	4340	27.6	999,718	12.4	< 0.0001	10,490	22.2	< 0.0001
Diabetes	1548	9.8	370,171	4.6	< 0.0001	4109	8.7	< 0.0001
Dyslipidemia	2747	17.5	790,913	9.8	< 0.0001	6927	14.7	< 0.0001
Ischemic Heart Disease	502	3.2	134,811	1.7	< 0.0001	1482	3.1	0.75
Cerebrovascular Disease	229	1.5	87,613	1.1	< 0.0001	808	1.7	0.03

We presented ORs for cardiovascular diseases of bus company employees compared to those of general workers in Table 5. In the crude model, ORs for five diseases were significantly higher in the bus company employee group. When we made the adjustment in the model with age groups, sex, income level quartiles, lack of exercise, smoking status, heavy drinking and obesity, ORs of hypertension (1.34, 95% CI: 1.29–1.40), diabetes (1.16, 95% CI: 1.10–1.22), dyslipidemia (1.18, 95% CI: 1.13–1.23) remained significantly higher. The OR of ischemic heart disease (1.00, 95% CI: 0.91–1.09) became non-significant, and the OR of cerebrovascular disease (0.81, 95% CI: 0.71–0.93) changed to be significantly lower. Finally, ORs for cardiovascular diseases of bus company employees compared to those of the propensity score matched controls were calculated and presented in Table 6. Bus company employees show significantly higher ORs for hypertension (1.33, 95% CI: 1.28–1.39), diabetes (1.14, 95% CI: 1.08–1.22), dyslipidemia (1.23, 95% CI: 1.17–1.29) compared to propensity score matched control group. The ORs for ischemic heart disease and cerebrovascular disease in the propensity score matched model were not significantly higher.

**Table 5:** Odds ratios for cardiovascular diseases of bus company employees compared to these of general workers

	Odds ratio (95%CI)			
	Crude model		Adjusted model^a^	
Hypertension	2.68	(2.59–2.78)	1.34	(1.29–1.40)
Diabetes	2.26	(2.15–2.39)	1.16	(1.10–1.22)
Dyslipidemia	1.94	(1.86–2.02)	1.18	(1.13–1.23)
Ischemic Heart Disease	1.93	(1.77–2.11)	1.00	(0.91–1.09)
Cerebrovascular Disease	1.34	(1.18–1.53)	0.81	(0.71–0.93)

^a^Adjusted for age groups, sex, income level quartiles, lack of exercise, smoking status, heavy drinking and obesity

**Table 6:** Odds ratios for cardiovascular diseases of bus company employees compared to those of propensity score matched controls

	Odds ratio (95%CI)	
Hypertension	1.33	(1.28–1.39)
Diabetes	1.14	(1.08–1.22)
Dyslipidemia	1.23	(1.17–1.29)
Ischemic Heart Disease	1.02	(0.92–1.13)
Cerebrovascular Disease	0.85	(0.73–0.98)

## Discussion

We found that bus company employees have higher odds of having hypertension, diabetes mellitus, and dyslipidemia when compared to general workers or propensity score matched controls. These results were consistent with the previous studies as bus drivers, who are the majority of bus company employees, have occupational risk factors of cardiovascular diseases [6, 9, 10]. The odds of having ischemic heart disease showed no statistically significant difference between two groups in the adjusted model and the propensity score matched model. The odds of bus company employees having cerebrovascular disease were lower than the general workers after adjusting the covariates, which was consistent with our results when we used the propensity score matched controls.

The mechanisms of these associations could be explained as follows. Stress in commercial bus driving may cause negative neurophysiological effects as driving can be conceptualized as a kind of threat avoidance task [25, 26]. Driving can actually promote cardiovascular risk markers. One’s blood pressure and pulse rate were elevated during driving [25]. We might assume that bus drivers may execute worse health behaviors. This assumption, however, was not supported in this study population as self-reported health behaviors like smoking, heavy drinking, and lack of exercise were more prevalent in the general population than bus drivers. The other reason we can consider is the fact that the average age was significantly higher in the bus driver group than that in the general population. Therefore, we used statistical adjustment of age groups and propensity score matching to compensate the vast effects of age on the target diseases’ prevalence.

In Korea, the working hours are legally limited to same or below 40 h per week, which can be extended up to 68 h when there is an agreement between the employee and the employer. In the certain industries, including bus transportation, exceptions exist at this regulation. The working hours can be extended up to any level with an agreement. There is a report that average daily working hours of bus drivers in the Korea are from 11 h to 18 h [17]. It has been studied that long working hours are associated with increased risks of cardiovascular diseases [27–30]. We cannot conclude this association in this study because there is no information of working hours. However, it is possible that long working hours in bus drivers could be additional burden to hypertension, diabetes, hyperlipidemia.

The insignificant relationship of ischemic heart diseases and the negative relationship of cerebrovascular diseases are partially due to the relatively severe consequences of the diseases. Those with history of these diseases have more likely worse health conditions and not capable of driving, especially special vehicles like bus. This may cause a kind of healthy worker effect, diminishing the current prevalence of the two disease groups [31]. Previous bus drivers who experienced ischemic heart disease and/or cerebrovascular disease might have not been able to return to work [32].

This study has several limitations. First, the Korean National Health Insurance data only contain the company information which a worker is registered. We could not infer a worker’s actual job characteristics based on the company’s information as workers may have various job characteristics in one company. Although most employees at the bus companies (> 90%) are bus drivers, we could not ensure the absence of bias caused by non-driving employees. Second, we could only use employees of bus companies in Seoul. As the number of employees in bus companies in Korea is estimated to be > 140,000 in 2015, 15,719 employees in Seoul may not sufficiently represent the whole industry [33]. Third, this is a cross-sectional designed study. Therefore, causal relationships between occupational risk factors among bus company employees and cardiovascular risks such as hypertension, diabetes, and dyslipidemia could not be inferred. We cannot conclude in the same context that the retirement of the bus company employees with previous ischemic heart disease and cerebrovascular disease caused the discordance of the results among the five diseases. A longitudinal study will be needed to overcome these limitations and to conclude causal relationships.

Despite these limitations, the present study has a few strong points. First, we could use the data of the whole working population of Korea. Although we inevitably excluded workers without general health check-up data, the number of the study participants were enough to represent the actual population. This fact enabled us to directly compare the actual disease prevalence between the two groups. Second, we utilized the propensity score matching technique to compensate the vast difference in base characteristics between bus company employee and general workers. The adjusted logistic regression model and the propensity score matched logistic regression model showed almost the same results. These two-way approaches support the reliability of results.

This study revealed that more intensive cardiovascular disease prevention measures for bus drivers and other bus company employees should be implemented to reduce the future risk of cardiovascular diseases. Some of the modifiable work-related risk factors, such as long working hours and cabin ergonomics, could be modulated also. Regular cardiovascular risk factor check-up for every bus company employee may be another effective measure.

To strengthen the evidence of this study’s findings, we suggest that further studies with longitudinal design should be conducted. Moreover, in the actual bus driver cohort, the dynamic occupational cohort that consisted of consecutive annual data of the Korean National Health Insurance can be established and analyzed to evaluate the causal relationship [31, 34].

## Conclusions

We evaluated the cardiovascular disease prevalence of bus drivers and compared the results to that of the general working population using the Korean National Health Insurance data. We found that the odds for having hypertension, diabetes, and dyslipidemia were significantly higher in the bus driver group. To determine the clear causal relationship, further studies with longitudinal design are needed.

## Abbreviations

BMI: Body Mass Index
KCD: Korean Classification of Diseases
NHI: National Health Insurance
OR: Odds ratio
